# Characterizing acute and persistent symptoms of COVID-19 among adults and children in rural Zambia, 2020–2022

**DOI:** 10.1371/journal.pgph.0006463

**Published:** 2026-05-28

**Authors:** Catherine G. Sutcliffe, Pamela Sinywimaanzi, Mutinta Hamahuwa, Juliet Morales, Adriana van de Guchte, Morris Sianyanda, Zain Khalil, Passwell Munachoonga, Ana Silvia Gonzalez-Reiche, Mathias Muleka, Katherine Z. J. Fenstermacher, Mwaka Monze, Harm van Bakel, Richard E. Rothman, Andrew Pekosz, Edgar Simulundu

**Affiliations:** 1 Department of Epidemiology, Johns Hopkins University Bloomberg School of Public Health‌‌, Baltimore, Maryland, United States of America; 2 Department of International Health, Johns Hopkins University Bloomberg School of Public Health, Baltimore, Maryland, United States of America; 3 Macha Research Trust, Choma, Zambia; 4 Department of Genetics and Genomic Sciences, Icahn School of Medicine at Mount Sinai‌‌, New York, New York, United States of America; 5 Department of Emergency Medicine, Johns Hopkins University School of Medicine, Baltimore, Maryland, United States of America; 6 Virology Laboratory, University Teaching Hospital, Lusaka, Zambia; 7 Department of Microbiology, Icahn School of Medicine at Mount Sinai, New York, New York, United States of America; 8 Department of Artificial Intelligence and Human Health, Icahn School of Medicine at Mount Sinai, New York, New York, United States of America; 9 Icahn Genomics Institute, Icahn School of Medicine at Mount Sinai, New York, New York, United States of America; 10 Department of Microbiology and Immunology‌‌, Johns Hopkins University Bloomberg School of Public Health, Baltimore, Maryland, United States of America; University of Antwerp, BELGIUM

## Abstract

Since emerging in December 2019, SARS-CoV-2 has led to significant morbidity and mortality globally. While much is known about SARS-CoV-2 infection and sequelae, fewer studies have been conducted in sub-Saharan Africa. This study was conducted to characterize acute and persistent symptoms of COVID-19 among adults and children in rural Zambia. From December 2020 to March 2022, individuals of all ages with acute SARS-CoV-2 infection (n = 132) were recruited into a cohort study. For comparison, individuals with acute influenza infection (n = 56) were also recruited. Participants were followed at 30, 90, 180, and 365 days. Most adult COVID-19 participants (median age: 36 years; 54% male; 11% had received the COVID-19 vaccine) were symptomatic at enrollment (92%), but few were hospitalized (9%). The most common symptoms were cough, headache, fever, and body aches. Most symptoms resolved by 30 days; the proportion reporting symptoms at follow-up visits ranged from 11% to 19%. Common symptoms at follow-up visits included cough, runny nose, headache, body aches, and fatigue. Similarly, most child COVID-19 participants (86%) were symptomatic at enrollment (median age: 13 years; 57% male; none had received the COVID-19 vaccine) and none were hospitalized. The most common symptoms were cough, fever, headache, and runny nose with most symptoms resolving by 30 days. The proportion reporting symptoms at follow-up visits ranged from 13% to 20%. Common symptoms included cough, fever, headache and runny nose. For comparison, adult and child participants with influenza virus infection were evaluated. The proportion reporting symptoms at follow-up visits ranged from 16% to 32%. This study adds to the literature on the clinical characteristics of acute SARS-CoV-2 infection and persistent symptoms in the first two years of the pandemic in an underrepresented region.

## Introduction

Since emerging in late 2019, over 777 million cases of COVID-19 and 7 million deaths have been reported worldwide, including over 9.5 million cases and 175,000 deaths in the African region [1]. Acute COVID-19 symptoms include fever, chills, cough, fatigue, myalgia, loss of taste or smell, and can range from mild to severe, with some infections leading to hospitalization or death, particularly for older adults and those with underlying medical conditions including HIV [2]. Following acute illness, an estimated 36% of individuals globally will experience post COVID-19 conditions (PCC; also known as long COVID or post-acute sequelae of COVID-19), although estimates from individual studies have ranged from 1% to 92% [3]. PCC is defined as symptoms usually occurring three months after acute COVID-19 and lasting for at least two months [4]. PCC can affect multiple organ systems and manifest in many ways, with common symptoms including memory problems, muscle weakness, breathlessness, general malaise, post-exertional malaise, post-traumatic stress disorder symptoms, joint pain, mood swings, and sleep problems [3,5]. Individuals with severe acute illness, have underlying medical conditions, have not received the COVID-19 vaccine, and females are at increased risk of PCC [3]. HIV may also increase the risk of PCC, given the chronic immune activation, inflammation and other comorbidities often associated with infection [6].

In the first months of the pandemic, when cases were concentrated in the Northern Hemisphere, dire predictions were made about the scale of COVID-19-related morbidity and mortality in the African region due to weaknesses in health infrastructure and the high prevalence of comorbidities including HIV, malaria, tuberculosis, and malnutrition [7,8], which raised concerns about the ability of countries to manage and control the outbreak [9]. In addition, much of the research infrastructure to evaluate COVID-19 was being established in the Northern Hemisphere. To address this, the Center for Excellence in Influenza Research and Surveillance (CEIRS) network leveraged existing studies of influenza virus to evaluate and characterize SARS-CoV-2 infections in the Southern Hemisphere. Participating centers established cohorts in May 2020 that included individuals with acute SARS-CoV-2 infection and a comparison group of individuals with acute influenza virus infection and followed participants using standardized protocols.

The Johns Hopkins CEIRS initiated influenza studies in rural Zambia in 2018 and participated in this Southern Hemisphere consortium. The first case of COVID-19 in Zambia was documented on March 18, 2020 [10] and mitigation measures were implemented soon thereafter, including screening and quarantine of travelers into Zambia, restrictions on travel for residents and public gatherings, closure of schools, indoor dining and recreational facilities, and mandatory mask-wearing in public spaces [11]. Restrictions were eased in phases starting in April 2020, with most restrictions lifted by September 2020. In July 2020, a serological survey in six districts found a prevalence of 10.6% for antibodies to SARS-CoV-2 [12]. This analysis provides results for the Johns Hopkins CEIRS cohort in rural Zambia and characterizes the acute and persistent symptoms of COVID-19 among adults and children from 2020-2022.

## Methods

### Ethics statement

This study was approved by the Johns Hopkins Institutional Review Board (IRB00168163), the Macha Research Trust Institutional Review Board (E.2018.02) and the Zambian National Health Research Authority. Adult participants and the parents or legal guardians of pediatric participants provided written informed consent.

### Study setting and population

The study was conducted in the catchment area of Macha Hospital, a rural area located in Southern Province, Zambia. The area is sparsely populated, with residents primarily involved in subsistence farming. In addition to mitigation measures implemented in March 2020, routine testing of patients and staff for SARS-CoV-2 was initiated at the hospital in May 2020, with contact tracing and testing triggered for positive cases. The first case of COVID-19 in the Macha area was identified in December 2020 [13].

### Study overview and procedures‌‌

The study was nested within existing respiratory surveillance activities initiated in December 2018 by the Johns Hopkins CEIRS and Macha Research Trust (MRT), as previously described [14–16]. Briefly, year-round surveillance for influenza-like illness (ILI; documented or reported fever plus cough or sore throat with onset or worsening in the past 7 days) was conducted in the outpatient department and the male, female, and pediatric wards. Patients were eligible if they had ILI, had not been enrolled in the past 30 days, and were willing to provide consent and contact information. Eligible patients were recruited for a study visit, which included administration of a questionnaire and collection of a nasopharyngeal swab. The nasopharyngeal swab was tested at the MRT Clinical Research Lab for influenza A/B virus and respiratory syncytial virus using the GeneXpert Xpress Flu/RSV assay (Cepheid, Sunnyvale, CA). From May 1, 2020 onwards, all samples were also tested for SARS-CoV-2, initially using various RT-qPCR kits (the Charité-Berlin protocol [17]; Da An Gene Co Ltd, Guangzhou, China [18]; and Maccura Biotechnology Co. Ltd., Chengdu, China [19]) provided by the Zambian Ministry of Health through the Zambia National Public Health Institute, and then using the GeneXpert SARS-CoV-2 assay (Cepheid, Sunnyvale, CA) when it became available.

All participants testing positive for SARS-CoV-2 through the Johns Hopkins CEIRS surveillance were recruited to participate in a cohort study. In addition, individuals testing positive for SARS-CoV-2 (with or without symptoms) through routine testing of patients and staff at the hospital and contact tracing were recruited for enrollment. As a comparison group, all Johns Hopkins CEIRS surveillance participants testing positive for influenza A/B virus were also recruited. After enrollment in the cohort, participants had a baseline visit and four follow-up visits at Day 30, 90, 180, and 365. At the baseline visit, a questionnaire was administered and a nasopharyngeal swab was collected (oropharyngeal swabs were offered as an alternative, but none were collected). For participants enrolled from Johns Hopkins CEIRS surveillance, the surveillance visit served as the baseline visit. At each follow-up visit, a questionnaire was administered.

### Laboratory procedures

An aliquot of the nasopharyngeal sample collected at the baseline visit was shipped to the Icahn School of Medicine at Mount Sinai for sequencing. SARS-CoV-2 genome sequencing was performed using a custom tiled genome amplification and assembly protocol [20,21]. Briefly, sequencing libraries were prepared from 1.5-2kb amplicons with the Nextera XT kit, run on the Illumina MiSeq platform in paired-end 2 × 150 nt format, and assembled using vRAPID (https://github.com/BakelLab/vRAPID). Viral genomes were then assigned to lineages with the Phylogenetic Assignment of Named Global Outbreak LINeages PANGO classification scheme and the Pangolin tool v. 3.1.17 [22].

For influenza viruses, whole-genomes were amplified with a multi-segment one-step RT-PCR as previously described [23], with custom primers for influenza A and B targeting the conserved sequences in the non-coding regions. Same as SARS-CoV-2, Nextera XT sequencing libraries were prepared for influenza A/B viruses and the Illumina MiSeq platform was used for sequencing under the same parameters. The assembled genomes were annotated using the API version of FLAN [24].

### Statistical analysis

Characteristics of participants, symptoms of the acute illness, and symptoms and health status during follow-up were summarized using descriptive statistics by age group (0–17 and ≥18 years) and separately for participants infected with SARS-CoV-2 and influenza virus. One participant had both SARS-CoV-2 and influenza B virus detected at enrollment and was grouped as SARS-CoV-2 for the purposes of analysis. Proportions were reported for categorical variables, and median and interquartile range (IQR) were reported for continuous variables. Comparisons between symptoms reported during the pre-Omicron and Omicron periods were performed using the chi-square test or Fisher’s exact test for categorical variables and the Wilcoxon rank-sum test for continuous variables. Given the small numbers of adults enrolled with influenza virus infection and children with SARS-CoV-2 infection, statistical comparisons between these groups were not made.

Latent Class Analysis was used to evaluate symptom clusters among participants infected with SARS-CoV-2 and correlates of class membership. The optimal number of classes was determined based on fit statistics. As the number of enrolled children was small, the analysis was restricted to adults.

Symptoms consistent with PCC were evaluated and defined for the purposes of this study as the presence of symptoms for at least two consecutive follow-up visits between Day 30 and 180. Risk factors for symptoms consistent with PCC were evaluated; due to small numbers, the proportion with symptoms consistent with PCC was compared between levels of covariates using Fisher’s exact tests.

Descriptive analyses and the Latent Class Analysis were conducted in SAS Software, Version 9.4 (SAS Institute Inc., Cary, North Carolina, USA). The alluvial plots of symptoms over time were created with R Foundation for Statistical Computing (Vienna, Austria), and the symptom class heat map was created with Stata Statistical Software, Version 14 (StataCorp, LLC., College Station, Texas, USA).

## Results

**Fig 1:**
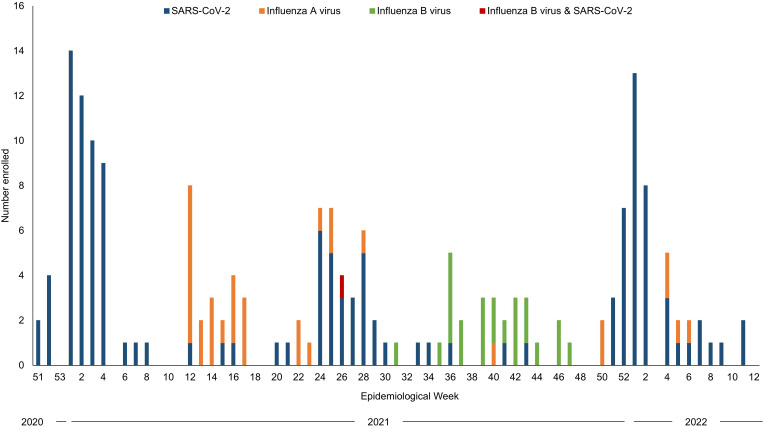
Enrollment into the study in Macha, Zambia, December‌‌ 2020 to March 2022.

From December 18, 2020 to March 31, 2022, 188 participants were enrolled, including 131 participants infected with SARS-CoV-2, 56 participants infected with influenza A/B virus, and one participant infected with both SARS-CoV-2 and influenza B virus (Fig 1; see Fig A and Fig B in S1 Appendix for enrollment for adults and children).

**Fig 2:**
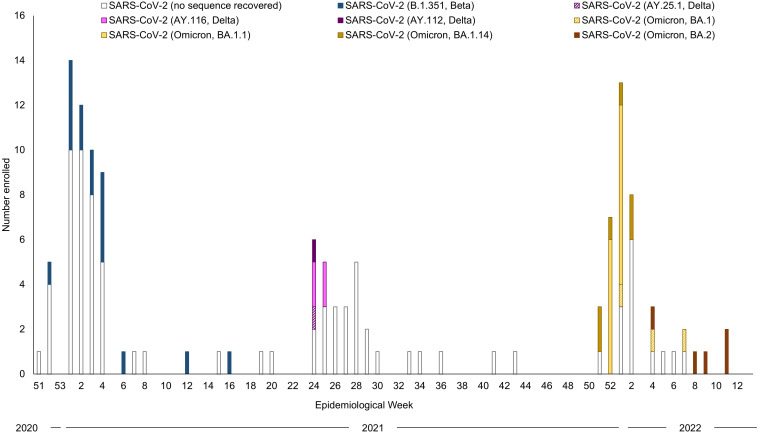
Sequencing results for participants infected with SARS-CoV-2 in Macha, Zambia, December 2020 to March 2022.

For SARS-CoV-2, enrollment peaked in January 2021, June 2021, and January 2022, corresponding to pandemic waves due to the Beta, Delta, and Omicron variants (Fig 2). Participants enrolled in the cohort were primarily adults (89.4%; Table 1).

**Table 1:** Characteristics of study participants with COVID-19 and influenza in Macha, Zambia, 2020-2022.

	Adults		Children	
	COVID-19 (n = 118)	Influenza (n = 12)	COVID-19)^a^ (n = 14)	Influenza (n = 44)
Male, n (%)	64 (54.2)	5 (41.7)	8 (57.1)	28 (63.6)
Age (years), median (interquartile range; range)	36 (28, 48; 18, 79)	29 (22, 44; 18, 58)	13 (7, 15; 0, 16)	4 (2, 8; 0, 13)
Age (years), n (%)				
0-4	–	–	2 (14.3)	25 (56.8)
5-17	–	–	12 (85.7)	19 (43.2)
18-49	90 (76.3)	10 (83.3)	–	–
50-64	23 (19.5)	2 (16.7)	–	–
≥65 years	5 (4.2)	0 (0.0)	–	–
Education, n (%)				
None	4 (3.4)	0 (0.0)	–	–
Basic/Primary	29 (24.6)	8 (66.7)	–	–
High/Secondary	43 (36.4)	3 (25.0)	–	–
College/University	42 (35.6)	1 (8.3)	–	–
Occupation, n (%)				
Healthcare provider	34 (28.8)	0 (0.0)	–	–
Teacher	9 (7.6)	0 (0.0)	–	–
Farmer	35 (29.7)	8 (72.7)	–	–
Other	40 (33.9)	3 (27.3)	–	–
Any underlying medical conditions, n (%)	31 (26.3)	3 (25.0)	1 (7.1)	0 (0.0)
# people living in household, median (IQR; range)	6 (4, 8; 1, 20)	7 (5, 8; 1, 18)	6 (5, 9; 4, 25)	6 (5, 8; 3, 27)
Other household members with respiratory symptoms in past 14 days, n (%)	65 (55.1)	6 (50.0)	10 (71.4)	25 (56.8)
Recent travel, n (%)				
Self	23 (19.5)	0 (0.0)	0 (0.0)	1 (2.3)
Others in household	16 (13.6)	0 (0.0)	1 (7.1)	1 (2.3)
Contact with known COVID-19 positive individual, n (%)	41 (34.8)	0 (0.0)	5 (35.7)	0 (0.0)
Received COVID vaccine before enrollment, n (%)^b^	12 (10.7)	0 (0.0)	0 (0.0)	0 (0.0)
Symptomatic at enrollment, n (%)	109 (92.4)	12 (100.0)	12 (85.7)	44 (100)
Days since symptom onset, median (Interquartile range: range)	5 (3, 7; 1, 14)	3 (3, 4; 1, 6)	4 (3, 6; 1, 7)	3 (3, 5; 1, 7)
Tachypnea, n (%)^c^	24 (22.2)	6 (50.0)	6 (50.0)	4 (9.1)
Hypoxemia, n (%)^d^	9 (8.3)	1 (8.3)	0 (0.0)	1 (2.3)
Sought care for illness, n (%)	87 (79.8)	12 (100.0)	9 (75.0)	44 (100.0)
Hospitalized for illness, n (%)	5 (8.8)	0 (0.0)	0 (0.0)	3 (7.3)

^a^ Includes the one participant co-infected with influenza B virus.

^b^ Among participants eligible for the vaccine.

^c^ Tachypnea defined by age: 0–1 months: > 60; 2–11 months: > 50; 1–4 years: > 40; 5–11 years: > 30; ≥ 12 years: 20.

^d^ Hypoxemia defined as oxygen saturation ≤92% measured at enrollment.

**Fig 3:**
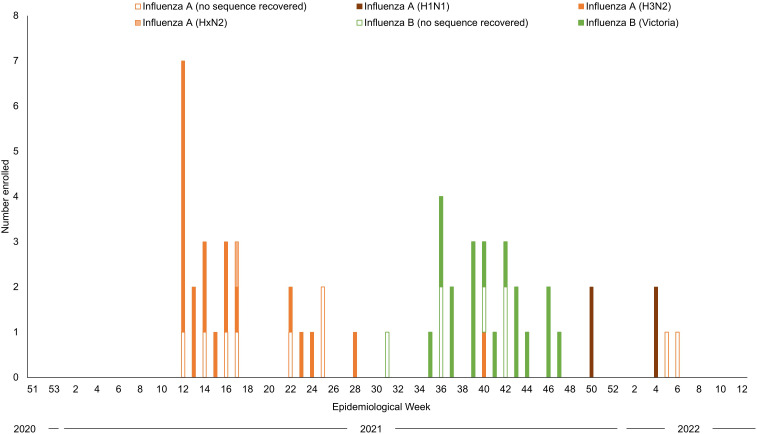
Sequencing results for participants infected with influenza virus in Macha, Zambia, December 2020 to March 2022.

For influenza virus, enrollment peaked in March/April 2021, August/September 2021, and December 2021/January 2022, corresponding to circulation of influenza A virus (H3N2), influenza B virus (Victoria lineage), and influenza A virus (H1N1), respectively (Fig 3). These patterns were similar to those from 2019 [25]. Participants enrolled in the cohort were primarily children (78.6%; Table 1).

### Characteristics of acute illness and persistence of symptoms among adults

Among adult COVID-19 participants (n = 118; Table 1), over half were male (54.2%), most had at least a high school education (72.0%), and almost a third were healthcare providers (28.8%) and had contact with a known COVID-19 positive person (34.8%). A quarter had an underlying medical condition (26.3%), with the most common being cardiovascular disease (17.1%), HIV (5.9%), and diabetes (5.1%), and 10.2% had received at least one dose of the COVID-19 vaccine (Janssen/Johnson & Johnson vaccine: n = 9; Oxford/AstraZeneca vaccine: n = 2; Pfizer-BioNTech vaccine: n = 1) prior to enrollment.

**Fig 4:**
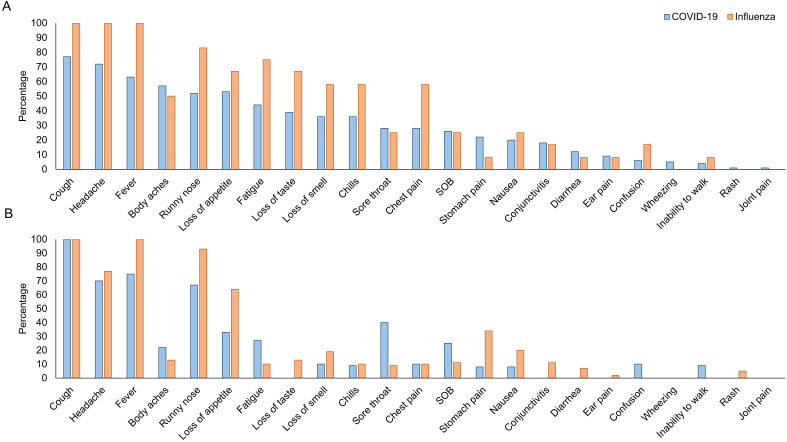
Self-reported symptoms at enrollment among symptomatic participants among A) adults and B) children. SOB: shortness of breath.

Most adult COVID-19 participants were symptomatic at enrollment (92.4%) with a median of 5 days since symptom onset (Table 1). Among symptomatic participants, most sought care (79.8%), and few were hospitalized (8.8%). Among adults who sought care, 82.0% reported receiving antibiotics; no participants received antivirals. Among the 56 participants receiving care at Macha Hospital and prescribed antibiotics, 50% received azithromycin, 20% received amoxicillin, 20% received cotrimoxazole, 5% received erythromycin, 3% received cefotaxime, and 2% received cefalexin. The most common symptoms were cough, headache, fever, and body aches (Fig 4; see Fig C in S1 Appendix for comparison of symptoms by variant period). Among symptomatic participants (n = 109), cluster analysis found that participants were equally distributed in two groups based on symptoms reported at enrollment (Fig D in S1 Appendix). One ‘severe’ group (n = 55) included participants experiencing many symptoms (median: 10; IQR: 8, 12), with more than half reporting cough, headache, fever, body aches, loss of appetite, changes in smell and taste, runny nose, fatigue, and chills. Most participants (94.6%) in this group had sought care for their illness, including all 5 adults who were hospitalized. A second ‘mild’ group (n = 54) included participants experiencing fewer symptoms (median: 5; IQR: 2, 6), with cough, headache, and fever the most common symptoms reported by more than half of the participants. Many participants (64.8%) had sought care for their illness, and none were hospitalized. Only sex was significantly associated with group membership, with females significantly more likely to be in the severe group (odds ratio: 2.9; 95% confidence interval: 1.2, 6.8).

**Fig 5:**
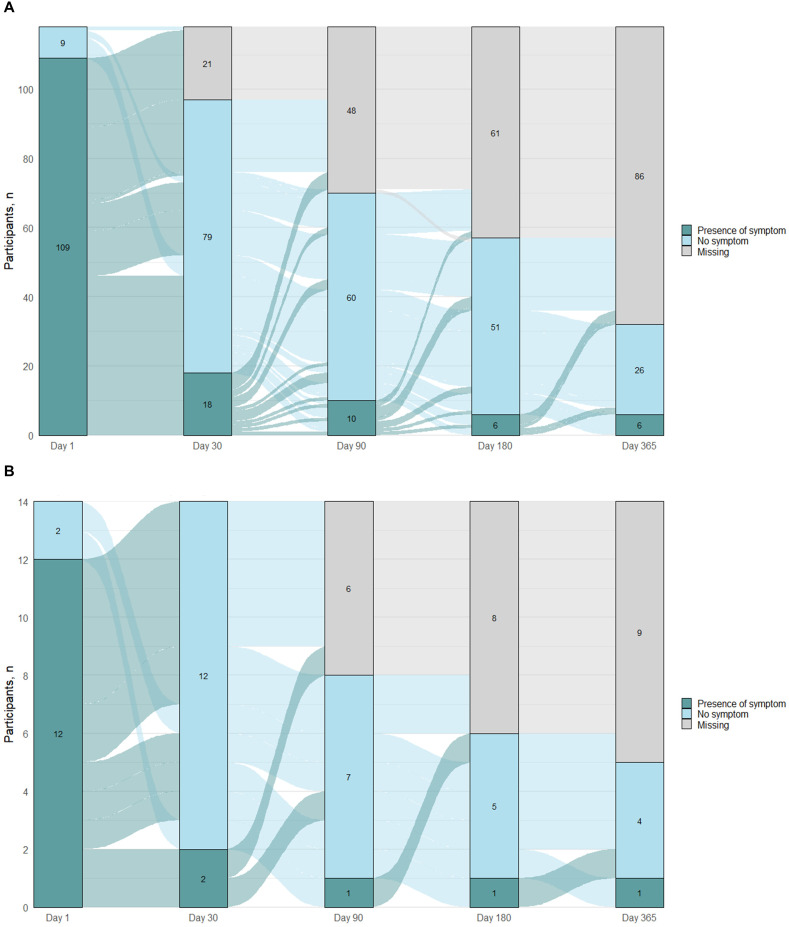
Presence of symptoms at enrollment‌‌ and follow-up among A) adults and B) children with COVID-19.

By 30 days after enrollment, most participants reported that their symptoms had resolved (Fig 5A;Fig E1 and Table B in S1 Appendix), with only 18.6% of adults reporting symptoms. A similar proportion of participants continued to report symptoms through 365 days, although the same participants did not report symptoms at each visit. Among adults, only 3/18 participants with symptoms at Day 30 continued to report symptoms at all available follow-up visits (5/18 had no additional visits; 5/18 reported no further symptoms; 5/18 reported symptoms at ≥1 other visit); 13 adults without symptoms at Day 30 reported symptoms at ≥1 other visit. The most common symptoms during follow-up were cough, runny nose, headache, body aches, and fatigue. Overall, 5.2% of participants (5/97) met the study definition for symptoms consistent with PCC. Older participants (13.0% for ≥50 years vs. 2.7% for 18–49 years of age; p = 0.08), participants hospitalized for their illness (33.3% vs. 4.9%; p = 0.20), and participants with underlying medical conditions (12.0% vs. 2.8%; p = 0.11) were more likely to have symptoms consistent with PCC, although these results were not statistically significant (Table C in S1 Appendix).

By 30 days after enrollment, most participants reported that they had returned to usual activities and that they were doing those activities as well as they had done them before (Fig E1 in S1 Appendix). Few participants reported financial hardship, that their relationships had suffered, or that they were more sad or depressed than before their illness.

For comparison, 12 adult participants with influenza virus were enrolled (Table 1). Most had a primary school education (66.7%; p = 0.03 for comparison with COVID-19 adult participants) and were farmers (72.7%; p = 0.03). A quarter had an underlying medical condition (25.0%; one each of HIV, kidney disease, chronic lung disease, and anemia). No influenza participants had received the COVID-19 vaccine. By study design, all participants were symptomatic at enrollment and sought care for their illness (91.7% received antibiotics; none received antivirals), although none were hospitalized. Participants were enrolled a median of 3 days after symptom onset (p = 0.007). Beyond symptoms of ILI, common symptoms were runny nose, headache, and fatigue (Fig 4A). By 30 days after enrollment, only 25.0% of participants reported symptoms and most had returned to usual activities (Table B and Fig E1 in S1 Appendix). Some adults (20.0-27.3%) continued to report symptoms through 180 days.

### Characteristics of acute illness and persistence of symptoms among children

Among child participants, over half were male (57.1%), a third (35.7%) had contact with a known COVID-19 positive person, one child had an underlying medical condition (tuberculosis; 7.7%), and none had received a dose of a COVID-19 vaccine. Most were symptomatic at enrollment (85.7%) with a median of 4 days since symptom onset (Table 1). Among symptomatic participants, most (75.0%) sought care, and none were hospitalized. Among children who sought care, 90.0% received antibiotics; none received antivirals. Among the 9 children receiving care at Macha Hospital and prescribed antibiotics, 44% received cotrimoxazole, 33% received amoxicillin, and 22% received azithromycin. The most common symptoms were cough, fever, headache, and runny nose (Fig 1).

By 30 days after enrollment, most participants reported that their symptoms had resolved (Fig 5B; Fig E2 and Table D in S1 Appendix), with only 14.3% of children reporting symptoms. A similar proportion of participants continued to report symptoms through 365 days, although the same participants did not report symptoms at each visit. Among children, 0/2 participants reporting symptoms at Day 30 reported symptoms at follow-up visits (one had no additional visits); three participants without symptoms at Day 30 each reported symptoms at one other visit. Symptoms during follow-up included cough, fever, headache and runny nose. No participants met the study definition for symptoms consistent with PCC.

By 30 days after enrollment, most participants reported that they had returned to usual activities and that they were doing those activities as well as they had done them before (Fig E2). At 30 days after enrollment, a small proportion of participants reported financial hardship, that their relationships had suffered, or that they were more sad or depressed than before their illness, with no participants reporting beyond 30 days.

For comparison, 44 child participants with influenza were enrolled (Table 1). They were significantly younger (median: 4 years) than children with COVID-19 (median: 13 years), and by study design, all were symptomatic at enrollment and sought care for their illness (95.5% received antibiotics; none received antivirals). Beyond symptoms of ILI, headache, runny nose, and loss of appetite were common (Fig 4B). By 30 days after enrollment, only 15.9% of children reported symptoms and most had returned to usual activities (Table D and Fig E2 in S1 Appendix). Some children (16.7-31.6%) continued to report symptoms through 365 days.

## Discussion

In this cohort of adults and children with acute SARS-CoV-2 infection in rural Zambia, most participants had a mild infection, with common symptoms of cough, headache, and fever. Most symptoms had resolved by 30 days, although 10–20% of both adults and children reported persistent symptoms at visits for up to 12 months, and a small proportion (5.2% of adults and no children) had symptoms consistent with PCC. This study adds to the literature on the clinical characteristics of acute SARS-CoV-2 infection and symptoms consistent with PCC in the first two years of the pandemic in an underrepresented region [3,6].

SARS-CoV-2 testing was initiated in Macha in May 2020, and despite many cases occurring throughout Southern Province and the country in the first few months of the pandemic [26], the first COVID-19 cases were only identified in December 2020. Macha is a rural area that is not easily accessible from major transportation routes and hubs. Consequently, contact with other areas may have been limited until most COVID-19 mitigation measures were lifted in September 2020 [13]. The first cases identified in Macha were enrolled in the cohort and included workers at the hospital, many of whom had a history of recent travel, suggesting that SARS-CoV-2 was imported into the area, spreading to hospital personnel and then quickly into the community. Enrollment peaked in January and June 2021 and again in January 2022. These peaks corresponded with the second, third, and fourth waves of the pandemic due to the Beta, Delta, and Omicron variants and are consistent in timing with peaks reported in Zambia and the region [1,27,28].

The clinical presentation of adults and children with mostly outpatient illness in this cohort was consistent with other studies in Africa [29,30], with the most common symptoms reported being cough, headache, and fever. In a study of all individuals admitted to COVID-19 care centers in West Africa, half had mild symptoms and half had moderate to severe symptoms, similar to the results of the symptom cluster analysis in this stud [30]. Similar to this study, many participants received antibiotics as treatment for COVID-19, including 85% who were prescribed azithromycin [30]. Azithromycin was a common treatment for COVID-19 in many regions, including Africa, often in combination with hydroxychloroquine, despite limited evidence to support its use [31]. While antibiotics are critical for treatment of secondary bacterial infections, which often follow viral respiratory infections, indiscriminate use of antibiotics can lead to selection for antimicrobial resistance, jeopardizing future treatment options and ongoing efforts to increase antibiotic stewardship [32].

The persistence of COVID-19 symptoms over the 12-month follow-up was also assessed, and although the WHO definition [4] for PCC could not be applied given the data collected, 5% of adults and no children reported symptoms at consecutive visits. Studies of PCC in Africa are limited, particularly among children [33,34]. Studies among adults in Africa have reported prevalence estimates ranging from 2% to 86% with follow-up periods mostly less than 6 months [33]. The range of symptoms, common symptoms, and increased proportion of persistent symptoms among older adults, adults with underlying medical conditions, and adults hospitalized for their illness in this study are consistent with other studies [33]. Studies among children globally have reported prevalence estimates ranging from 1.8% to 70% [34]. In a study of children at a COVID-19 treatment center in Ethiopia with mostly asymptomatic or mild illness, 4.6% reported persistent symptoms through 3 months of follow-up [35]. Given the small number of children in this cohort, the finding that 12.5% of children reported symptoms at 3 months and that no children met the study definition for PCC is consistent with these studies.

In this study, a comparison group of individuals with influenza virus infection was enrolled, but the limited overlap in the age distribution with individuals with SARS-CoV-2 infection made comparisons challenging. However, the finding of persistent symptoms in 16–32% of children and adults is consistent with other studies that enrolled a non-COVID-19 comparison group with symptoms of acute respiratory illness [36,37], and provides support for the occurrence of these symptoms following other viral and non-viral acute illnesses.

This study was subject to limitations. This was a cohort study that recruited participants through a variety of mechanisms, including at the health facility and in the community. Given the challenges of recruiting patients in the COVID-19 ward of the hospital and with the most severe symptoms, it is likely that adults and children with severe illness are underrepresented. The size of the cohort was relatively small, which limited the assessment of risk factors for severe illness, symptoms consistent with PCC, and comparisons between participants infected with SARS-CoV-2 and influenza virus. This was particularly true for children, which reflects the predominance of symptomatic and medically-attended illness among adults [38], as well as challenges in recruiting children attending boarding schools in the area who were identified through contact tracing. The assessment of symptom persistence and symptoms consistent with PCC was limited by the follow-up schedule and data collection instruments. This study was designed in May 2020, when much was still unknown about acute SARS-CoV-2 infection and PCC was only beginning to be recognized [39]. A standard protocol was developed that could be implemented by all participating centers, and data collection forms were modeled after other initiatives at the time. While information about PCC evolved, the forms remained the same, which preserved comparability of the data over time, but limited the application of updated case definitions. Lastly, this was a cohort study conducted in one rural area of Zambia. The results should be interpreted in this context and should not be interpreted to represent the experience of all individuals with SARS-CoV-2 infection in this area or throughout Zambia and the region.

In summary, this cohort study documented the clinical characteristics of acute SARS-CoV-2 infection and symptoms consistent with PCC among adults and children in rural Zambia. The findings highlight the range of symptoms experienced during the acute illness and the persistence of symptoms in up to 20% of individuals after infection with both SARS-CoV-2 and other respiratory pathogens. This study adds to the literature on COVID-19 and respiratory infections in a region that is underrepresented in research studies and surveillance.

## Supporting information

S1 Appendix Supporting Information. (DOCX)

S1 Checklist Inclusivity in global research. (DOCX)
