# Supplementary material for: Characterizing acute and persistent symptoms of COVID-19 among adults and children in rural Zambia, 2020–2022
Source: PLOS Glob Public Health. 2026 May 28;6(5):e0006463. doi: 10.1371/journal.pgph.0006463 (PMC13218479; doi:10.1371/journal.pgph.0006463)
Supplement: S1 Appendix — (DOCX) [file pgph.0006463.s001.docx]

**Supporting Information**

**Characterizing acute and persistent symptoms of COVID-19 among adults and children in rural Zambia, 2020-2022**

Catherine G. Sutcliffe, Pamela Sinywimaanzi, Mutinta Hamahuwa, Juliet Morales, Adriana van de Guchte, Morris Sianyanda, Zain Khalil, Passwell Munachoonga, Ana Silvia Gonzalez-Reiche, Mathias Muleka, Katherine Z. J. Fenstermacher, Mwaka Monze, Harm van Bakel, Richard E. Rothman, Andrew Pekosz, Edgar Simulundu

**Table A. Correlates of symptom group membership among adults with COVID-19 in Macha, Zambia, 2020-2022**

|  | **Membership in severe symptom group**  **N (%)** | **Unadjusted Odds Ratio**  **(95% Confidence Interval)** | **Adjusted Odds Ratio**  **(95% Confidence Interval)** |
| --- | --- | --- | --- |
| Sex |  |  |  |
| Male | 23 (39.7) | Ref | Ref |
| Female | 32 (62.8) | 2.8 (1.2, 6.6) | 2.9 (1.2, 6.8) |
| Age (years) |  |  |  |
| 50-64 | 43 (52.4) | Ref | Ref |
| ≥65 years | 12 (44.4) | 0.8 (0.3, 2.1) | - |
| Any underlying medical conditions |  |  |  |
| No | 37 (46.3) | Ref | Ref |
| Yes | 18 (62.1) | 2.0 (0.8, 5.0) | 2.1 (0.8, 5.5) |
| Received COVID vaccine before enrollment |  |  |  |
| No | 49 (50.0) | Ref | Ref |
| Yes | 6 (54.6) | 1.3 (0.3, 4.7) | - |
| Hospitalized for illness |  |  |  |
| No | 50 (48.1) | Ref | Ref |
| Yes | 5 (100) | - | - |
| Variant period^a^ |  |  |  |
| Pre-Omicron | 36 (49.3) | Ref | Ref |
| Omicron | 19 (52.8) | 1.2 (0.5, 2.9) | - |

^a^ Omicron period start defined as December 1, 2021 based on available sequencing data.

**Table B. Self-reported symptoms at enrollment and follow-up among participants ≥18 years of age in Macha, Zambia, 2020-2022**

|  | COVID-19 | | | | | Influenza | | | | |
| --- | --- | --- | --- | --- | --- | --- | --- | --- | --- | --- |
|  | Day 1 (n=118) | Day 30 (n=97) | Day 90 (n=70) | Day 180 (n=57) | Day 365 (n=32) | Day 1  (n=12) | Day 30 (n=12) | Day 90 (n=11) | Day 180 (n=5) | Day 365 (n=1) |
| Any symptoms | 109 (92.4) | 18 (18.6) | 12 (17.1) | 6 (10.5) | 6 (18.8) | 12 (100) | 3 (25.0) | 3 (27.3) | 1 (20.0) | 0 |
| Cough | 84 (77.1) | 11 (61.1) | 5 (41.7) | 2 (33.3) | 6 (100.0) | 12 (100) | 2 (66.7) | 1 (33.3) | 0 | - |
| Headache | 78 (71.6) | 5 (27.8) | 7 (58.3) | 2 (33.3) | 2 (33.3) | 12 (100) | 2 (66.7) | 1 (33.3) | 0 | - |
| Fever | 68 (63.0)^a^ | 0 | 2 (16.7) | 0 | 2 (33.3) | 12 (100) | 1 (33.3) | 0 | 0 | - |
| Body aches | 62 (57.4)^a^ | 3 (16.7) | 2 (16.7) | 1 (16.7) | 2 (33.3) | 6 (50.0) | 0 | 0 | 0 | - |
| Loss of appetite | 58 (53.2) | 1 (5.6) | 2 (16.7) | 0 | 2 (33.3) | 8 (66.7) | 0 | 0 | 0 | - |
| Runny nose | 57 (52.3) | 7 (38.9) | 4 (33.3) | 4 (66.7) | 1 (16.7) | 10 (83.3) | 3 (100.0) | 1 (33.3) | 0 | - |
| Fatigue | 48 (44.0) | 4 (22.2) | 2 (16.7) | 1 (16.7) | 1 (16.7) | 9 (75.0) | 1 (33.3) | 0 | 1 (20.0) | - |
| Loss of taste | 42 (38.5) | 1 (5.6) | 2 (16.7) | 1 (16.7) | 1 (16.7) | 8 (66.7) | 0 | 0 | 0 | - |
| Loss of smell | 39 (36.1)^b^ | 1 (5.6) | 0 | 1 (16.7) | 1 (16.7) | 7 (58.3) | 1 (33.3) | 0 | 0 | - |
| Chills | 39 (35.8) | 2 (11.1) | 1 (8.3) | 0 | 1 (16.7) | 7 (58.3) | 0 | 0 | 0 | - |
| Chest pain | 31 (28.4) | 2 (11.1) | 2 (16.7) | 1 (16.7) | 1 (16.7) | 7 (58.3) | 0 | 1 (33.3) | 1 (20.0) | - |
| Sore throat | 30 (27.5) | 1 (5.6) | 4 (33.3) | 0 | 2 (33.3) | 3 (25.0) | 0 | 0 | 0 | - |
| Shortness of breath | 28 (25.7) | 1 (5.6) | 0 | 0 | 0 | 3 (25.0) | 0 | 0 | 0 | - |
| Stomach pain | 23 (21.5)^a^ | 1 (5.6) | 0 | 0 | 1 (16.7) | 1 (8.3) | 1 (33.3) | 1 (33.3) | 0 | - |
| Nausea | 22 (20.2) | 0 | 0 | 1 (16.7) | 1 (16.7) | 3 (25.0) | 0 | 1 (33.3) | 0 | - |
| Conjunctivitis | 20 (18.4) | 0 | 0 | 1 (16.7) | 0 | 2 (16.7) | 0 | 0 | 0 | - |
| Diarrhea | 13 (11.9) | 0 | 1 (8.3) | 1 (16.7) | 1 (16.7) | 1 (8.3) | 1 (33.3) | 0 | 0 | - |
| Ear pain | 10 (9.2) | 0 | 0 | 0 | 1 (16.7) | 1 (8.3) | 0 | 0 | 0 | - |
| Confusion | 7 (6.4) | 0 | 0 | 0 | 0 | 2 (16.7) | 1 (33.3) | 0 | 0 | - |
| Wheezing | 5 (4.6) | 0 | 1 (8.3) | 0 | 0 | 0 | 0 | 1 (33.3) | 0 | - |
| Inability to walk | 4 (3.7) | 1 (5.6) | 1 (8.3) | 0 | 0 | 1 (8.3) | 0 | 0 | 0 | - |
| Rash | 1 (0.9) | 0 | 1 (8.3) | 0 | 0 | 0 | 0 | 0 | 0 | - |

^a^ Missing for one participant

^b^ Missing for two participants

**Table C. Risk factors for post-COVID conditions among participants ≥18 years of age in Macha, Zambia, 2020-2022**

|  | **PCC^a^**  **N (%)** | **p-value^b^** |
| --- | --- | --- |
| Sex |  | 0.39 |
| Male | 4 (7.1) |  |
| Female | 1 (2.4) |  |
| Age (years) |  | 0.08 |
| 18-49 | 2 (2.7) |  |
| ≥50 years | 3 (13.0) |  |
| Any underlying medical conditions |  | 0.11 |
| No | 2 (2.8) |  |
| Yes | 3 (12.0) |  |
| Received COVID vaccine before enrollment |  | 1.00 |
| No | 5 (5.6) |  |
| Yes | 0 (0.0) |  |
| Hospitalized for illness |  | 0.20 |
| No | 2 (4.9) |  |
| Yes | 1 (33.3) |  |
| Variant period^a^ |  | 0.61 |
| Pre-Omicron | 3 (4.2) |  |
| Omicron | 2 (7.7) |  |

^a^ Post-COVID condition (PCC) defined as the presence of symptoms for at least two consecutive follow-up visits between Day 30 and 180.

^b^ p-value from Fisher’s exact test

**Table D. Self-reported symptoms at enrollment and follow-up among participants <18 years of age in Macha, Zambia, 2020-2022**

|  | COVID-19 | | | | | Influenza | | | | |
| --- | --- | --- | --- | --- | --- | --- | --- | --- | --- | --- |
|  | Day 1  (n=14) | Day 30 (n=14) | Day 90 (n=8) | Day 180 (n=6) | Day 365 (n=5) | Day 1  (n=44) | Day 30 (n=44) | Day 90 (n=27) | Day 180 (n=19) | Day 365 (n=6) |
| Any symptoms | 12/14 (85.7) | 2/14 (14.3) | 1/8 (12.5) | 1/6 (16.7) | 1/5 (20.0) | 44/44 (100) | 7/44 (15.9) | 7/27 (25.9) | 6/19 (31.6) | 1/6 (16.7) |
| Cough | 12/12 (100) | 1/2 (50.0) | 0 | 0 | 0 | 44/44 (100) | 5/7 (71.4) | 5/7 (71.4) | 5/6 (83.3) | 0 |
| Fever | 9/12 (75.0) | 0 | 1/1 (100.0) | 0 | 0 | 44/44 (100) | 0 | 2/7 (28.6) | 3/6 (50.0) | 0 |
| Headache | 7/10 (70.0) | 0 | 0 | 0 | 0 | 17/22 (77.3) | 1/7 (14.3) | 1/7 (14.3) | 4/6 (66.7) | 1/1 (100.0) |
| Runny nose | 8/12 (66.7) | 0 | 0 | 1 (100.0) | 1 (100.0) | 40/43 (93.0) | 4/7 (57.1) | 5/7 (71.4) | 3/6 (50.0) | 0 |
| Sore throat | 4/10 (40.0) | 0 | 0 | 0 | 0 | 2/22 (9.1) | 0 | 1/7 (14.3) | 0 | 0 |
| Loss of appetite | 4/12 (33.3) | 0 | 0 | 0 | 0 | 28/44 (63.6) | 2/7 (28.6) | 1/7 (14.3) | 0 | 0 |
| Fatigue | 3/11 (27.3) | 0 | 0 | 0 | 0 | 2/21 (9.5) | 0 | 0 | 0 | 0 |
| Shortness of breath | 3/12 (25.0) | 0 | 0 | 0 | 0 | 5/44 (11.4) | 0 | 1/7 (14.3) | 0 | 0 |
| Body aches | 2/9 (22.2) | 0 | 0 | 0 | 0 | 2/16 (12.5) | 0 | 0 | 0 | 0 |
| Chest pain | 1/10 (10.0) | 0 | 0 | 0 | 0 | 2/20 (10.0) | 0 | 0 | 0 | 0 |
| Loss of smell | 1/10 (10.0) | 0 | 0 | 0 | 0 | 3/16 (18.8) | 0 | 0 | 0 | 0 |
| Confusion | 1/10 (10.0) | 0 | 0 | 0 | 0 | 0/34 | 0 | 0 | 0 | 0 |
| Chills | 1/11 (9.1) | 0 | 0 | 0 | 0 | 2/21 (9.5) | 0 | 0 | 0 | 0 |
| Stomach pain | 1/12 (8.3) | 0 | 0 | 0 | 0 | 10/29 (34.5) | 0 | 0 | 1/6 (16.7) | 0 |
| Nausea | 1/12 (8.3) | 0 | 0 | 0 | 0 | 9/44 (20.5) | 0 | 1/7 (14.3) | 0 | 0 |
| Inability to walk | 1/11 (9.1) | 0 | 0 | 0 | 0 | 0/42 | 0 | 0 | 0 | 0 |
| Loss of taste | 0/10 | 0 | 0 | 0 | 0 | 2/16 (12.5) | 0 | 0 | 0 | 0 |
| Wheezing | 0/12 | 0 | 0 | 0 | 0 | 0/44 | 0 | 0 | 0 | 0 |
| Diarrhea | 0/12 | 0 | 0 | 0 | 0 | 3/44 (6.8) | 0 | 1/7 (14.3) | 1/6 (16.7) | 0 |
| Conjunctivitis | 0/12 | 0 | 0 | 0 | 0 | 5/44 (11.4) | 0 | 0 | 0 | 0 |
| Rash | 0/12 | 0 | 0 | 0 | 0 | 2/44 (4.6) | 0 | 0 | 0 | 0 |
| Ear pain | 0/12 | 0 | 0 | 0 | 0 | 1/43 (2.3) | 0 | 0 | 0 | 0 |

**Fig A. Study flowchart**

25 SARS

-

CoV

-

2 positive

patients identified through

ILI surveillance*

64 IAV/IBV positive

patients identified

through ILI surveillance

56 IAV/IBV positive

patients enrolled in the

cohort study

•

8 declined

132 SARS

-

CoV

-

2 positive

patients enrolled in the

cohort study

163 SARS

-

CoV

-

2 positive

patients identified through

MoH

testing

•

1

declined

•

6 excluded (lived outside study area)

•

49 declined

24 consented

108 consented

•

70 from symptomatic testing program

•

29 from contact tracing program

•

4 from occupational exposure

•

5 from staff routine testing program

Follow

-

up visits:

•

Day 30: n=111

•

Day 90: n=78

•

Day 180: n=63

•

Day 365: n=37

Follow

-

up visits:

•

Day 30: n=56

•

Day 90: n=38

•

Day 180: n=24

•

Day 365: n=7

IAV: influenza A virus; IBV: influenza B virus; ILI: influenza-like illness; MoH: Ministry of Health

*Includes one participant who tested positive for SARS-CoV-2 and influenza B virus

**Fig B. Enrollment into the study of 1) adults and 2) children in Macha, Zambia, December 2020 to March 2022**

**1)**

**2)**

**Fig C. Distribution of symptoms at enrollment among symptomatic adults with COVID-19, by variant period, 2020-2022**

*p<0.05 for comparison between pre-Omicron and Omicron period by chi-square or Fisher’s exact test

**Fig D. Symptom clustering at enrollment among symptomatic adults with COVID-19 in Macha, Zambia, 2020-2022**

**Fig E. Health status over time among 1) adults and 2) children with COVID-19 and influenza in Macha, Zambia, 2020-2022**

**1)**

**2)**
